# Safety and efficacy of probiotics in the treatment of ALD

**DOI:** 10.1097/MD.0000000000043662

**Published:** 2025-08-08

**Authors:** Xiangyu Zhou, Shuxi Zhou

**Affiliations:** a Department of Rehabilitation Medicine, Second Affiliated Hospital of Chengdu Medical College and 416 Hospital of Nuclear Industry, Chengdu, Sichuan, China.

**Keywords:** alcoholic liver disease, effectiveness, intestinal flora, meta-analysis, probiotics, security

## Abstract

**Background:**

The incidence of alcoholic liver disease (ALD) in China has been increasing in recent years, causing serious socioeconomic and public health burden. However, the treatment of ALD lacks a clear, unified, and effective plan. However, there is still lack of systematic analysis and evaluation of the efficacy and safety of probiotic in the treatment of ALD.

**Methods:**

A computerized search was performed to identify randomized controlled trials of probiotic and combined probiotic therapy in alcohol-induced liver injury. The control group received placebo or conventional treatment. Retrieval period from database creation to January7, 2022. Both investigators independently searched literature, extracted data, and assessed risk of bias using RevMan 5.4 software and rated the quality of the level of evidence for the outcome indicators according to the GRADE criteria evaluation.

**Results:**

Altogether 10 studies with 835 patients were included. Meta-analysis showed significantly better liver function in test group than control group. The inflammatory level in the experimental group was significantly lower than that in the control group. The intestinal flora of the experimental group was effectively improved. But only *Bifidobacteria* and *Lactobacilli* were indigenous; meta-analysis showed that the adverse reactions in the experimental group were slightly higher than those in the control group, but the results were insignificant. Current evidence shows that compared with conventional therapy, probiotic or probiotic combination therapy can effectively improve liver function, modulate the gut microbiota environment, and reduce inflammation in alcoholic liver by modulating the gut microbiota, but the safety needs further investigation. There are limitations in the quantity and quality of the included trials.

**Conclusion:**

This study shows that the use of probiotics therapy has a good regulating effect on liver function, intestinal flora, inflammatory level, and blood lipid level in ALD patients.

## 1. Introduction

Alcoholic liver disease (ALD) is a series of liver diseases caused by long-term chronic intake of alcohol leading to hepatocellular damage, and its development is divided into 5 disease processes, including alcoholic fatty liver, alcoholic steatohepatitis, alcoholic liver fiber, alcoholic cirrhosis, and liver cancer.^[1]^ At present, the alcohol consumption in China is growing in parallel with the economic growth, and the prevalence of ALD has gradually approached that of alcohol-abusing countries in Europe and the US,^[2]^ which has caused a serious burden on social economy and public health.

Long-term chronic alcohol intake leads to alterations in intestinal flora^[3]^ and impairs intestinal mucosal integrity and barrier function,^[4]^ increases intestinal permeability, translocates intestinal bacteria, and lipopolysaccharide enters the portal circulation, activates kuff cells as well as TOLL-like 4 receptors,^[5]^ and induces inflammatory and chemokine production. Probiotics play an important role in regulating the intestinal flora, and it has been shown that probiotic supplementation can repair the intestinal barrier, improve liver injury, and reduce inflammatory factor production.^[6–8]^ However, there has been no systematic evaluation of the efficacy of probiotics and probiotic combinations related to the treatment of ALD. Therefore, this study was conducted to systematically evaluate the published clinical randomized controls for the treatment of ALD through probiotics and probiotic combination medication in order to provide a reference for the treatment of ALD.

## 2. Information and methods

### 2.1. Inclusion and exclusion criteria

#### 2.1.1. Study type

Published domestic and international randomized controlled trial related to the application of probiotics and the combination of probiotics.

#### 2.1.2. Study subjects

Patients with clinically diagnosed ALD, including alcoholic fatty liver, alcoholic hepatitis, alcoholic liver fibrosis, and alcoholic cirrhosis, race, gender, and age were not restricted and the diagnostic criteria were according to the Guidelines for the Prevention and Treatment of ALD (2018 updated version).^[1]^

#### 2.1.3. Interventions

The test group used probiotics or probiotic combination, with no restriction on the method of administration, dose, or duration of treatment. The control group used placebo treatment or conventional treatment, with no limitation on the method of administration, dose or duration of treatment.

#### 2.1.4. Outcome indicators

Primary outcome indicators: liver function, alanine aminotransferase (ALT), aspartate aminotransferase (AST), and γ-glutamyl transferase (GGT), secondary outcome indicators: inflammatory factors high-sensitivity C-reactive protein (h-CRP), interleukin-1β (IL-1β), interleukin-6 (IL-6), tumor necrosis factor-α (TNF-α), intestinal flora: *Escherichia coli*, *Bifidobacterium*, *Lactobacillus*, *Enterococcus*, *Bacteroidetes*, total bilirubin (TBIL), total cholesterol (TC), and adverse reactions.

#### 2.1.5. Exclusion criteria

(1) Outcome indicators were not effectively available; (2) duplicate literature; (3) studies for which full text was not available; (4) reviews, conferences, animals, and in vitro studies.

### 2.2. Literature search strategy

Computer searches of CNKI, WanFang Data, VIP, PubMed, Web of Science, EMbase, and The Cochrane Library databases were conducted to collect randomized controlled trials of probiotics and probiotic combinations for the treatment of ALD, all from the time of database creation. The search was conducted using a combination of subject terms and free terms. The search terms included: ALDs, gastrointestinal microbiome, probiotic, randomized controlled trial, etc.

### 2.3. Literature screening and data extraction

Literature and data extraction were performed independently by evaluators and cross-checked, and in case of disagreement, a third party was consulted to assist with resolution. Literature screening was done first by reading the titles and abstracts of the articles to exclude literature that was clearly irrelevant to the current study, and then further by reading the full text to determine the final literature screening was done first by reading the titles and abstracts of the articles to exclude literature that was clearly irrelevant to the current study, and then further by reading the full text to determine the final literature to be included. If needed, the original authors of the literature were contacted by e-mail to obtain important information related to the current study. Included: (1) basic study information: title of the literature, first author, and year of publication of the literature; (2) baseline characteristics of the study population: sample size, intervention, and duration of intervention for each group of patients; (3) relevant outcome indicators.

### 2.4. Risk of bias evaluation of included studies

The included literature was evaluated for risk of bias according to the Cochrane Evaluation Manual,^[9]^ which included the method of random assignment, whether the method of assignment was hidden, whether double-blind trials were conducted, whether the data results had integrity, whether there was a potential for selective reporting of studies, and whether there were other sources of bias. After reading the full text, the literature was evaluated based on the above criteria and classified as low risk of bias, high risk of bias, and uncertain risk of bias based on the quality of the evaluation.

### 2.5. Assessment of the quality of evidence of included studies

We used the GRADE system, an online tool for assessing the quality of evidence for outcome indicators in the included literature, to assess the quality of evidence for risk of bias, inconsistency, accuracy, indirectness, and publication bias.

### 2.6. Statistical analysis

Meta-analysis was performed using RevMan 5.4 software. Relative risk was used as an effect indicator for the count data and mean difference (MD) was used as an effect indicator for the measure data, and point estimates and 95% confidence interval (CI) were given for each effect. If there was no statistical heterogeneity (I^2^ ≤ 50%) between the results of the studies, meta-analysis was performed using a fixed-effects model; if there was statistical heterogeneity (I^2^ ≥ 50%) between the results of the studies, the source of heterogeneity was further analyzed, and meta-analysis was performed using a random effects model after excluding the effect of obvious clinical heterogeneity. Obvious clinical heterogeneity was handled by methods such as subgroup analysis or sensitivity analysis, or only descriptive analysis was performed.

### 2.7. Literature screening process and results

**Figure 1.**
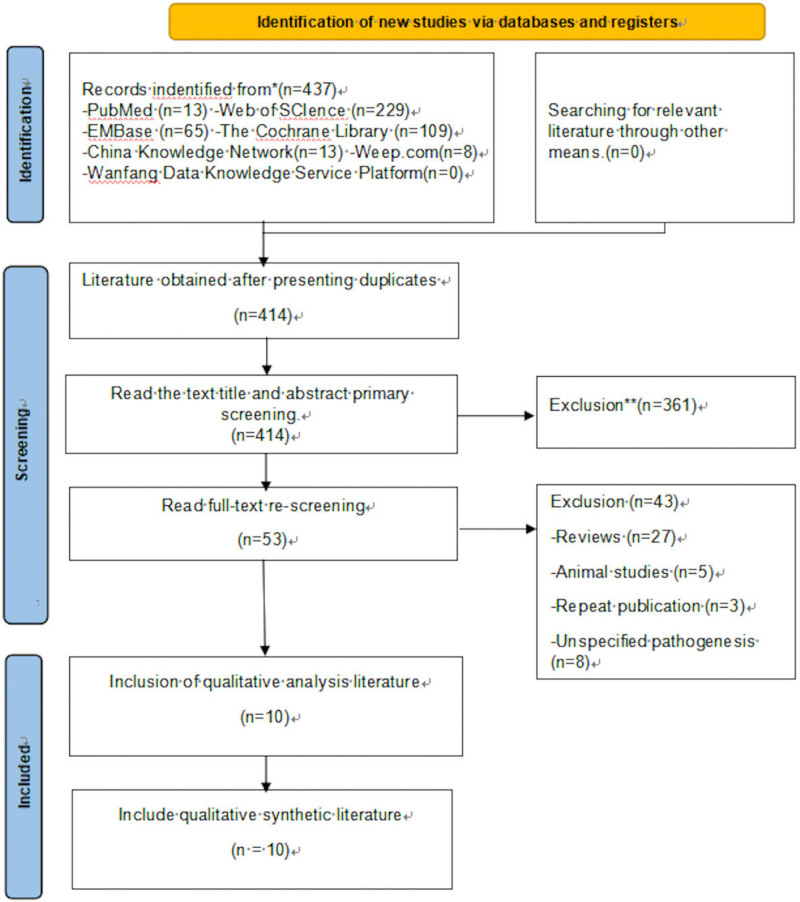
Literature screening process and results. The process of literature search, evaluation, exclusion, and inclusion is shown. A total of 21 research reports were fnally included, involving 1037 participants.

A total of 437 relevant papers were found through the search, 414 after excluding duplicates, 53 were included after reading the title and abstract, 43 were excluded after reading the full text, and finally 10 randomized controlled trials were included.^[10–19]^ The literature screening process and results are shown in Figure 1.

### 2.8. Basic characteristics of the included studies

The 10 included studies were all trials conducted in Asia from 2008 to 2021 and included 835 patients. Seven of the studies were conducted in China^[13–19]^ and the remaining 3 were conducted in Russia, Japan, and South Korea, respectively. Seven of the studies were conducted in China,^[10]^ and the remaining 3 were conducted in Russia, Japan,^[11]^ and South Korea,^[12]^ respectively. The basic characteristics of the included literature are specified in Table 1.

**Table 1:** Basic characteristics of included studies.

Inclusion in the study	Publish time	Age T/C	Region	Number of cases T/C	Observation group treatment measures	Control group treatment measures	Treatment	Observation of included outcome indicators
Irina A. Kirpich^[10]^	2008	42.16 ± 1.89/ 42.06 ± 2.18	Russia	32/34	Probiotics	Vitamin B1 + Vitamin B6 + Diazepam	12 wk	1, 2, 3, 5, 10, 11, 12, 13
Hironori Koga^[11]^	2013	52.6 ± 11.8/ 53.9 ± 14.9	Japan	18/19	Y400	Placebo	4 wk	1, 2, 3, 4, 6, 9, 10, 11, 12, 15
Sang Hak Han^[12]^	2015	52.7 ± 11.3/ 52.7 ± 11.3	Korea	60/57	Probiotics	Placebo	1 wk	1, 2, 3, 4, 5, 7, 8, 10, 11
Xiaohong Zhu^[13]^	2018	47.2 ± 3.2/ 48.3 ± 3.5	China	30/30	Montelukastan combined with *Bifidobacterium trifolium* tablets combined with Ginseng and Atractylodes	Montmorillonite combined with *Bifidobacterium* triethiodis tablets	8 wk	1, 3, 10, 11, 12, 13, 14, 15
Hui Zhu^[14]^	2019	47.2 ± 5.1/ 46.9 ± 5.2	China	40/40	*Clostridium typhimurium* tablets combined with polyunsaturated phosphatidylcholine capsules	Basic treatment	12 wk	1, 2, 5, 8, 9, 10, 11, 12, 13
Jianji Zhang^[15]^	2020	47.43 ± 4.22/ 46.72 ± 3.98	China	49/49	*Bifidobacterium tetrakis* tablets combined with montelukast	Basic treatment	12 wk	1.2.3.6.8.9. 10.11.12.13.15
Yuxia Zhou^[16]^	2020	49.24 ± 4.87/ 48.63 ± 5.04	China	48/48	*Bifidobacterium tetrakis* tablets	Basic treatment + montelukast	4 wk	1.2.3.10. 11.13
Xuelong Li^[17]^	2021	49.6 ± 4.17/ 52.60 ± 5.67	China	54/46	Beverages containing LcS bacteria cells	Fermented milk drinks (ordinary lactic acid drinks without Lcs bacteria)	8 wk	1, 2, 3, 4, 5, 7, 8, 9, 10, 11, 12, 13, 14
Jianji Zhang^[18]^	2021	46.0 ± 45.22/ 45.27 ± 5.09	China	39/40	Reduced glutathione Glycopeptide combined with *Bifidobacterium* tetradecanoides tablets	Reduced glutathione combined with *Bacillus subtilis* dibacterium enteric coated capsules	4 wk	1, 2, 3, 10, 11, 12, 13, 15
Chang Wu^[19]^	2021	55.3 ± 5.4/ 55.3 ± 5.4	China	51/51	Thiopronin tablets combined with *Clostridium typhimurium* live tablets	Thiopronine tablets in combination with metadoxine	12 wk	7, 8, 9, 10, 11, 12, 13

*Note*: 1. alanine aminotransferase (ALT); 2. aspartate aminotransferase (AST); 3. γ-glutamyl transferase (GGT); 4. total cholesterol (TC); 5. total bilirubin (TBIL); 6. high-sensitivity C-reactive protein (h-CRP); 7. interleukin-1β (IL-1β); 8. tumor necrosis factor-α (TNF- α); 9. interleukin-6 (IL-6); 10. *E coli*; 11. *Bifidobacterium*; 12. *Lactobacillus*; 13. *Enterococcus*; 14. *Bacteroides*; 15. adverse effects.

The 10 included studies were all trials conducted in Asia from 2008 to 2021 and included 835 patients. Seven of these studies were conducted in China,^[13–19]^ and the remaining 3 were conducted in Russia,^[10]^ Japan,^[11]^ and Korea.^[12]^

### 2.9. Evaluation of risk of bias and quality of the included literature

**Figure 2.**
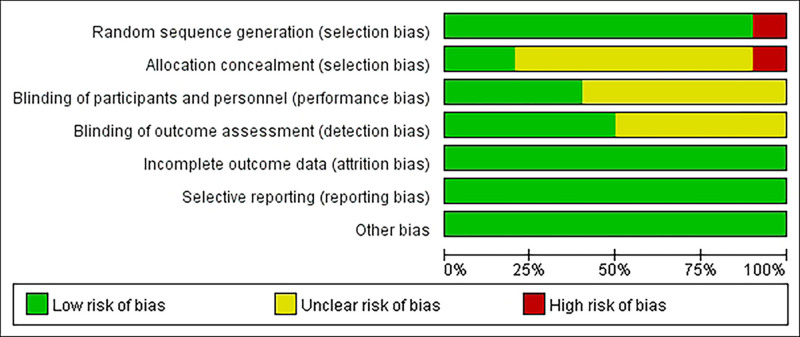
Risk bias evaluation. A total of 21 studies that met the criteria were fnally included in the meta-analysis. Three of the study participants were children, and one of the study participants had coexisting type 2 diabetes; 2 of the studies were not explicitly blinded, and one study was not randomized concealed.

**Figure 3.**
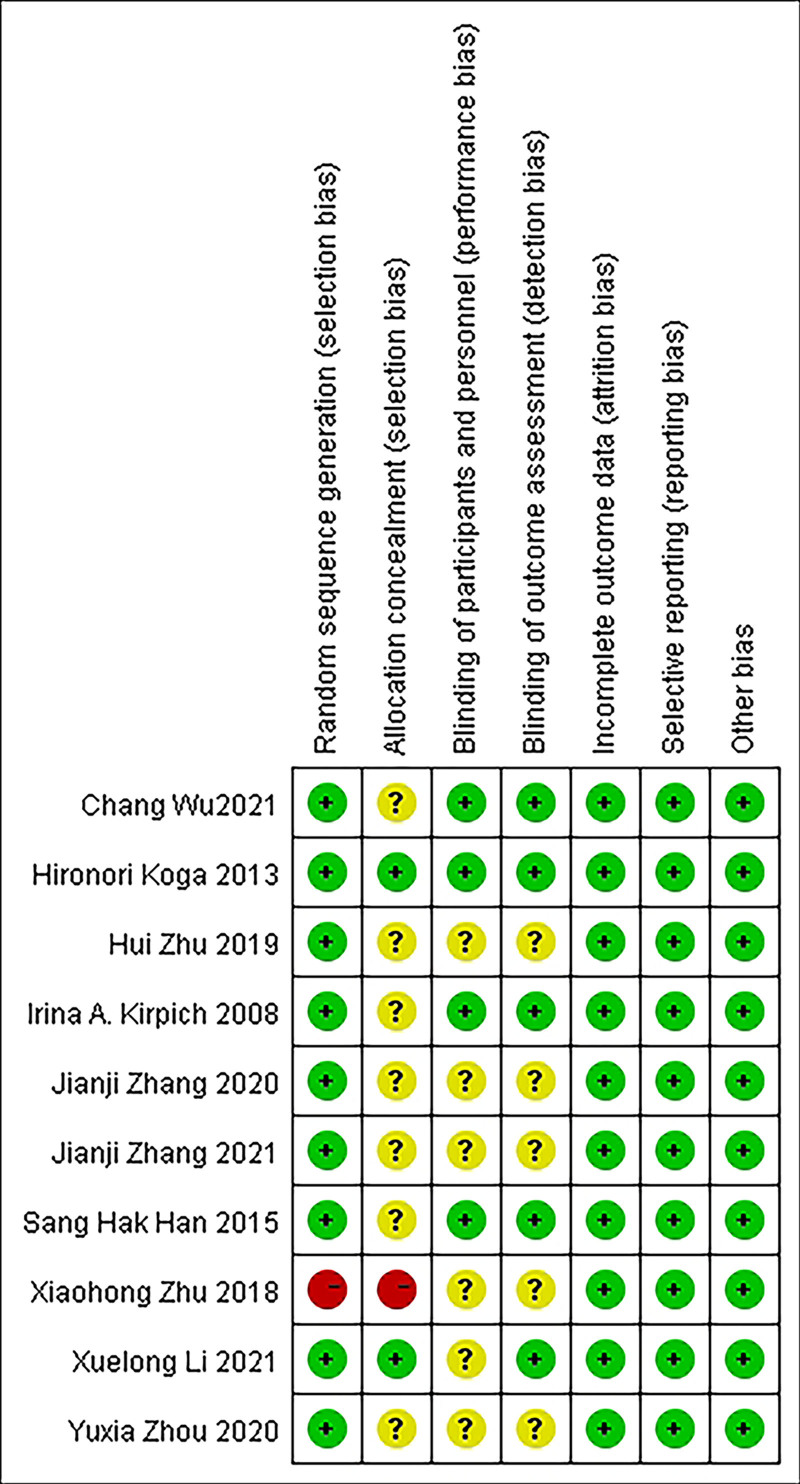
Summary of risk bias. A total of 21 studies that met the criteria were finally included in the meta-analysis. Three of the study participants were children, and one of the study participants had coexisting type 2 diabetes; 2 of the studies were not explicitly blinded, and 1 study was not randomized concealed.

Nine of all included studies in the literature used random assignment methods,^[10–12,14–19]^ of which 1^[10]^ used the random label method, 1 did not specify the randomization method,^[12]^ 2^[11,17]^ used the computerized randomization method, 4^[15,16,18,19]^ used the random number table method, and 1^[14]^ used coin flip method; all studies did not explicitly state allocation concealment; 3 studies^[11,12,17]^ explicitly used the double-blind method and the remaining all studies did not explicitly state whether blinding was performed; all studies had completeness of data results; all studies had no selective reporting. The quality evaluation table of the included studies is shown in Table 2, and the risk of bias evaluation of the included studies is shown in Figures 2 and 3.

**Table 2:** Quality evaluation of included studies methodology.

Num	Author	Publish time	Random assignment method	Allocation plan hidden	Blindness	Data result integrity	Selective reporting studies	Other sources of bias
No. 1	Irina A. Kirpich^[10]^	2008	Yes	No	Yes (double blind)	Yes	No	No
No. 2	Hironori Koga^[11]^	2013	Yes	No	Yes (double blind)	Yes	No	No
No. 3	Sang Hak Han^[12]^	2015	Yes	No	Yes (double blind)	Yes	No	No
No. 4	Xiaohong ZHU^[13]^	2018	Yes	No	No	Yes	No	No
No. 5	Hui Zhu^[14]^	2019	Yes	No	No	Yes	No	No
No. 6	Jianji Zhang^[15]^	2020	Yes	No	No	Yes	No	No
No. 7	Yuxia Zhou^[16]^	2020	Yes	No	No	Yes	No	No
No. 8	Xuelong Li^[17]^	2021	Yes	No	Yes (double blind)	Yes	No	No
No. 9	Jianji Zhang^[18]^	2021	Yes	No	No	Yes	No	No
No. 10	Chang Wu^[19]^	2021	Yes	No	No	Yes	No	No

*Note*: 10 studies were included in the study methodological quality assessment.

## 3. Meta-analysis results

### 3.1. ALT

**Figure 4.**
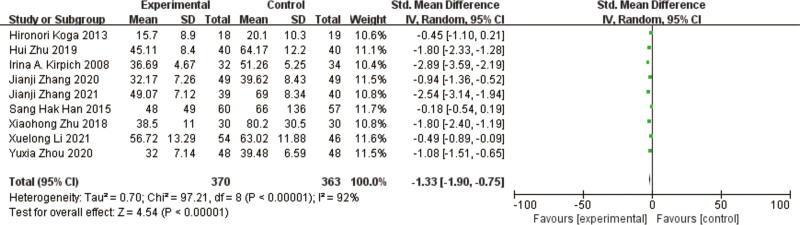
Forest plot of ALT comparison between experimental group and control group. A total of 9 studies reported the mean change in ALT from baseline. The results of the analysis showed that ALT levels were signifcantly reduced after probiotic intervention. ALT = alanine aminotransferase.

A total of 9 studies^[10–18]^ reported ALT in a total of 733 patients. The results of the heterogeneity test between the studies showed a large heterogeneity (*P* < .00001, I² = 92%), using a random effects model. The results of the meta-analysis showed that ALT was significantly lower in the test group compared with the control group, and the difference in the results was significant [MD = -1.33, 95% CI [-1.91, -0.76], *P* < .00001] (Fig. 4).

The heterogeneous results among the studies were large and in order to find out the reasons for the heterogeneity, it was found that after excluding the studies of Irina A et al,^[10]^ Xiaohong Zhu et al,^[13]^ Hui Zhu et al,^[14]^ Jianji Zhang et al,^[18]^ the heterogeneous results were low, I²  =  0%, *P* = .87, indicating that the homogeneity among the studies was good and the results of meta-analysis showed that the ALT in the test group was improved compared with the control group, and the difference in the results was significant [MD = -7.07, 95% CI [-8.89, -5.25], *P* < .00001].

### 3.2. AST

**Figure 5.**
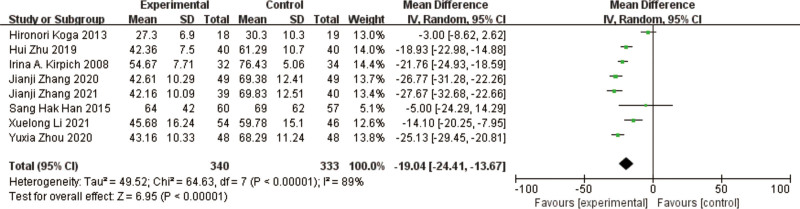
Forest plot of AST comparison between experimental and control group. A total of 8 studies reported the mean change from baseline in AST: the analysis showed that AST levels were signifcantly reduced after probiotic intervention. The results were signifcantly different. AST = aspartate aminotransferase.

A total of 8 studies^[10–12,14–18]^ reported AST in a total of 673 patients. The results of the heterogeneity test between studies showed a large heterogeneity (*P* < .00001, I² = 89%), using a random effects model. The results of the meta-analysis showed a significant reduction in AST in the treated trial group compared to the treated control group, with a significant difference in the results [MD = -19.04, 95% CI [-24.41, -13.67], *P* < .00001] (Fig. 5).

The results of heterogeneity among the studies were large, and to find out the reason for heterogeneity, by excluding the comparison one by one, it was found that after excluding the studies of Hironori Koga et al,^[11]^ Sang Hak Han et al,^[12]^ Hui Zhu et al,^[14]^ and Xuelong Li et al,^[17]^ the results of heterogeneity were low, I² = 45%, *P* = .14, indicating that the studies were the homogeneity was good, and the results of meta-analysis showed that AST improved in the test group compared to the control group, and the difference in results was significant [MD = -24.49, 95% CI [-26.53, -22.46], *P* < .00001].

### 3.3. GGT

**Figure 6.**
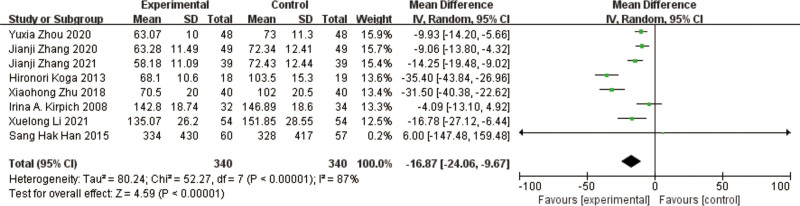
Forest plot of GGT comparison between experimental and control group. A total of 8 studies reported the mean change from baseline in GGT: the analysis showed that GGT levels were signifcantly reduced after probiotic intervention. The results were signifcantly different. GGT = γ-glutamyl transferase.

A total of 8 studies^[10–13,15–18]^ reported GGT in a total of 680 patients. The results of the heterogeneity test between studies showed a large heterogeneity (*P* < .00001, I² = 87%), using a random effects model. Meta-analysis showed that GGT was significantly lower in the test group compared to the control group, with a significant difference in the results [MD = -16.87, 95% CI [-24.06, -9.67], *P* < .00001] (Fig. 6).

The results of heterogeneity among the studies were large, and to find out the reasons for heterogeneity, by excluding the comparison one by one, it was found that Hironori Koga et al^[11]^ and Sang Hak Han et al,^[12]^ Zhu Xiaohong et al,^[13]^ 3 studies had better homogeneity, I² = 0%, *P* = .6, and the results of meta-analysis showed that GGT was improved in the test group compared to the control group, and the difference in results was significant [MD = -33.48, 95% CI [-39.60, -27.37], *P* < .00001].

### 3.4. h-CRP

**Figure 7.**
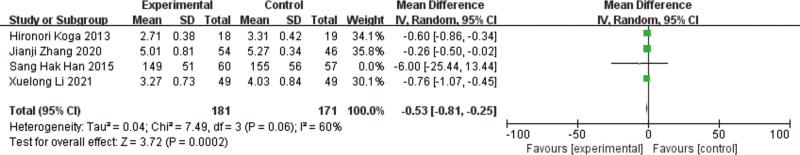
Forest plot of h-CRP comparison between experimental and control group. A total of 6 studies reported the mean change from baseline in h-CRP: the analysis showed that h-CRP levels were signifcantly reduced after probiotic intervention. The results were signifcantly different. h-CRP = high-sensitivity C-reactive protein.

A total of 4 studies^[11,12,15,17]^ reported h-CRP in a total of 352 patients. The results of the heterogeneity test between the studies showed a large heterogeneity (*P* = .06, I² = 60%), using a random effects model. Meta-analysis showed that h-CRP was reduced in the test group compared to the control group, with a significant difference in the results [*MD* = -0.53, 95% CI [-0.81, -0.25], *P* = .0002] (Fig 7).

The heterogeneous results among studies were large, to find out the reason for the heterogeneity, by excluding each comparison, it was found that after excluding Xuelong Li,^[17]^ the heterogeneous results were lower (I² = 0%, *P* = .64). The results of meta-analysis showed that h-CRP improved in the test group compared to the control group, and the difference in results was significant [MD = -0.67, 95% CI [-0.86, −0.47], *P* < .00001.

### 3.5. IL-6

**Figure 8.**
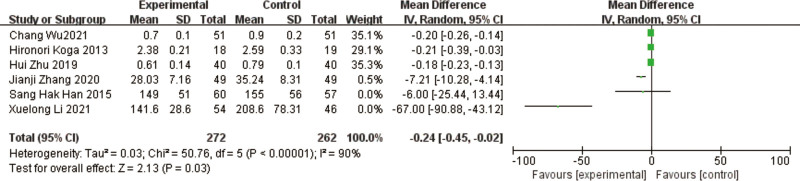
Forest plot of IL-6 comparison between experimental group and control group. A total of 6 studies reported the mean change from baseline in IL-6: the analysis showed that IL-6 levels were signifcantly reduced after probiotic intervention. The results were signifcantly different. IL-6 = interleukin-6.

It was reported in a total of 6 studies,^[11,12,14,15,17,19]^ for a total of 534 patients. The results of the heterogeneity test between studies showed a high heterogeneity (*P* < .00001, I² = 90%), using a random effects model. The results of the meta-analysis showed a decrease in IL-6 in the test group compared to the control group, with a significant difference in the results [MD = -0.24, 95% CI [-0.45, -0.02], *P* = .03] (Fig. 8).

The results of heterogeneity among studies were large, and to find out the reason for heterogeneity, by excluding one by one comparison, it was found that after excluding Jianji Zhang,^[15]^ Xuelong Li,^[17]^ the results of heterogeneity were lower (I² = 0%, *P* = .89), and the results of meta-analysis showed that IL-6 improved in the test group compared to the control group, and the difference in results was significant [MD = -0.19, 95% CI [-0.23, -0.15], *P* < .00001].

### 3.6. IL-1β

**Figure 9.**
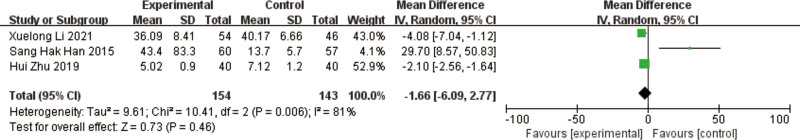
Forest plot of IL-1β comparison between experimental and control group. A total of 3 studies reported the mean change from baseline in IL-1β: the analysis showed that IL-1β levels were signifcantly reduced after probiotic intervention. The results were signifcantly different. IL-1β = interleukin-1β.

A total of 3 studies^[12,14,17]^ reported IL-1β in a total of 297 patients. The results of the heterogeneity test between studies showed a large heterogeneity (*P* = .006, I² = 81%), using a random effects model. The results of the meta-analysis showed that IL-1β was reduced in the test group compared to the control group, with a nonsignificant difference in the results [MD = -1.66, 95% CI [-6.09, 2.77], *P* = .46] (Fig. 9).

The heterogeneous results among the studies were large and to find out the reason for the heterogeneity, by excluding one by one comparison, it was found that after excluding SangHak Han et al^[12]^ the heterogeneous results were lower (I² = 41%, *P* = .19) and the results of meta-analysis showed that IL-1β improved in the test group compared to the control group, and the difference in the results was significant [MD = -2.53, 95% CI [-4.13, −0.93], *P* = .002].

### 3.7. TNF-α

**Figure 10.**
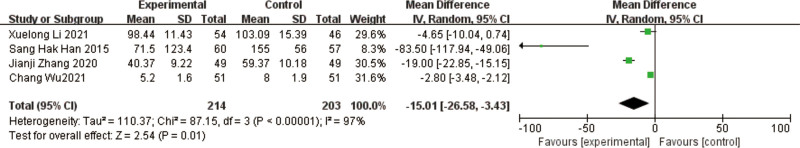
Forest plot of TNF-α comparison between experimental and control group. A total of 4 studies reported the mean change from baseline in TNF-α: the analysis showed that TNF-α levels were signifcantly reduced after probiotic intervention. The results were signifcantly different. TNF-α = tumor necrosis factor-α.

A total of 4 studies^[12,15,17,19]^ reported TNF-α in a total of 417 patients. The results of the heterogeneity test between the studies showed a large heterogeneity (*P* < .00001), *I²* = 97%), using a random effects model. The results of the meta-analysis showed that TNF-α was reduced in the treated trial group compared with the control group, and the difference in results was significant [MD = -15.01, 95% CI [-26.58, −3.43], *P* = .01] (Fig. 10).

The results of heterogeneity among studies were large, and in order to identify the causes of heterogeneity, comparisons were made by excluding one by one, but none of them were able to improve the heterogeneous results.

### 3.8. TBIL

**Figure 11.**
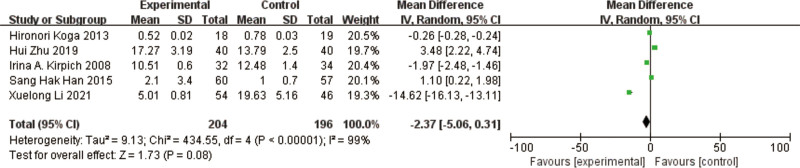
Forest plot of TBIL comparison between experimental and control group. A total of 5 studies reported the mean change from baseline in TBIL: the analysis showed that TBIL levels were signifcantly reduced after probiotic intervention. The results were signifcantly different. TBIL = total bilirubin.

A total of 5 studies^[10–12,14,17]^ reported TBIL, in a total of 400 patients. The results of the heterogeneity test between studies showed a large heterogeneity (*P* < .00001, I² = 99%), using a random effects model. The results of the meta-analysis showed a reduction in TBIL in the test group compared to the control group, with a nonsignificant difference in the results [MD = -2.37, 95% CI [-5.06, 0.31, *P* = .08] (Fig. 11).

The heterogeneous results among the studies were large, and to find the cause of the heterogeneity, comparisons were made by exclusion one by one, but none of them were able to improve the heterogeneous results.

### 3.9. E coli

**Figure 12.**
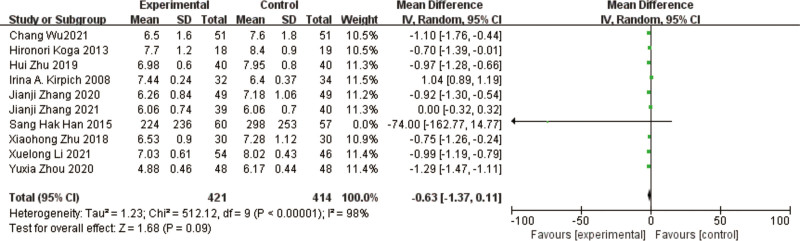
Forest plot of *E coli* comparison between experimental and control group. A total of 10 studies reported the mean change from baseline in E coli: the analysis showed that *E coli* levels were signifcantly reduced after probiotic intervention. The results were signifcantly different.

A total of 10 studies^[10–19]^ reported *E coli* in a total of 835 patients. The results of the heterogeneity test between studies showed a large heterogeneity (*P* < .00001, I² = 98%), using a random effects model. The results of the meta-analysis showed a reduction in the abundance of *E coli* in the test group compared to the control group, with a nonsignificant difference in the results [MD = -0.63, 95% CI [-1.37, 0.11], *P* = .09] (Fig. 12).

The heterogeneous results among the studies were large, and to find out the reasons for the heterogeneity, by excluding the studies of Irina A et al^[10]^, Zhang Jianji et al,^[18]^ the heterogeneous results were found to be low (I² = 44%, *P* = .09), and the results of meta-analysis showed that *E coli* improved in the test group compared to the control group, and the difference in the results was significant [MD = -1.02, 95% CI [-1.20, -0.84], *P* < .00001].

### 3.10. Bifidobacteria

**Figure 13.**
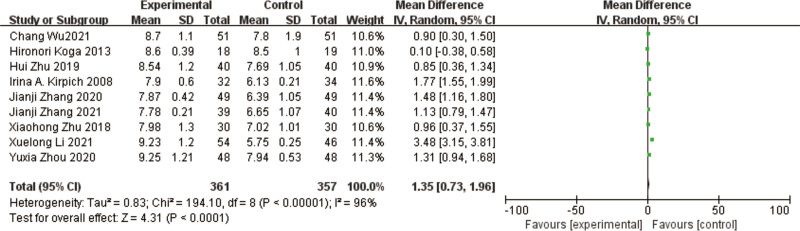
Forest plot of *Bifidobacteria* between experimental group and control group. A total of 9 studies reported the mean change from baseline in *Bifidobacteria*: the analysis showed that *Bifidobacteria* levels were signifcantly reduced after probiotic intervention. The results were signifcantly different.

A total of 9 studies^[10,11,13–19]^ reported bifidobacteria in a total of 718 patients. The results of the heterogeneity test between studies showed a large heterogeneity (*P* < .00001, I² = 96%) using a random effects model. The results of the meta-analysis showed that the abundance of bifidobacteria was increased in the test group compared to the control group, with a significant difference in the results [MD = 1.35, 95% CI [0.73, 1.96], *P* < .0001] (Fig. 13).

The heterogeneous results among the studies were large, and to find out the reason for the heterogeneity, by excluding the studies of Irina A et al,^[10]^ Hironori Koga et al,^[11]^ Xuelong Li et al,^[17]^ the heterogeneous results were low (I² = 29%, *P* = .22) and the results of meta-analysis showed that the bifidobacteria in the test group improved compared to the control group, and the difference in the results was significant [MD = 1.17, 95% CI [0.97, 1.38], *P* < .00001].

### 3.11. Lactobacillus

**Figure 14.**
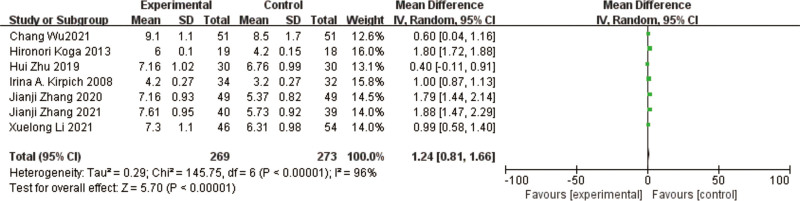
Forest plot of *Lactobacillus* comparison between experimental and control group. A total of 7 studies reported the mean change from baseline in *Lactobacillus*: the analysis showed that *Lactobacillus* levels were signifcantly reduced after probiotic intervention. The results were signifcantly different.

A total of 7 studies^[10,11,14,15,17–19]^ reported on *Lactobacillus*, for a total of 542 patients. The results of the heterogeneity test between studies showed a large heterogeneity (*P* < .00001, I² = 96%), using a random effects model. The results of the meta-analysis showed an increase in the abundance of *Lactobacillus* in the treated test group compared to the control group, with a significant difference in the results [MD = 1.24, 95% CI [0.81, 1.66], *P* < .0001] (Fig. 14).

The heterogeneous results among the studies were large, and to find out the reason for the heterogeneity, by excluding the comparison one by one, it was found that the heterogeneous results of the 3 studies Hironori Koga et al,^[11]^ Jianji Zhang,^[15]^ JianjiZhang et al^[18]^ were low (I² = 0%, *P* = .93), and the results of meta-analysis showed that *Lactobacillus* improved in the test group compared to the control group, and the difference in the results was significant [MD = 1.80, 95% CI [1.72, 1.88], *P* < .00001]. Besides, Irina A et al,^[10]^ Xuelong Li et al,^[17]^ Wu Chang et al,^[19]^ 3 studies were better homogeneous, I² = 0%, *P* = .39, meta-analysis showed that *Lactobacillus* improved in the test group compared to the control group and the difference in the results was significant [MD = 0.98, 95% CI [0.86, 1.10], *P* < .00001].

### 3.12. Enterococci

**Figure 15.**
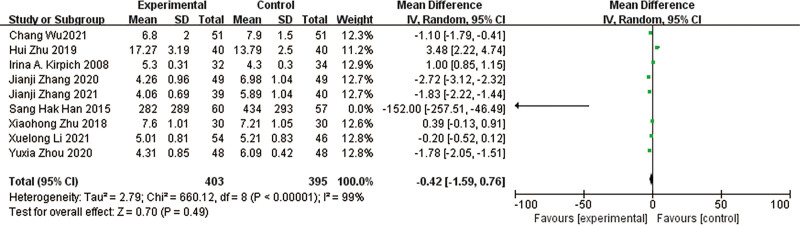
Forest plot of *Enterococci* comparison between experimental group and control group. A total of 9 studies reported the mean change from baseline in *Enterococci*: the analysis showed that *Enterococci* levels were signifcantly reduced after probiotic intervention. The results were signifcantly different.

A total of 9 studies^[10,12–19]^ reported enterococci in a total of 542 patients. The results of the heterogeneity test between studies showed a large heterogeneity (*P* < .00001, I² = 99%), using a random effects model. The results of the meta-analysis showed a decrease in the abundance of enterococci in the test group compared to the control group, with a nonsignificant difference in the results [MD = -0.42, 95% CI [-1.59, 0.76], *P* = .49] (Fig. 15).

The heterogeneous results among the studies were large, and to find out the reason for the heterogeneity, by excluding the comparison one by one, it was found that the heterogeneous results of 2 studies, Yuxia Zhou,^[16]^ Zhang Jianji et al,^[18]^ were low, I² = 0%, *P* = .84, and the results of meta-analysis showed that enterococci improved in the test group compared to the control group, and the difference in the results was significant [MD = -1.80, 95% CI [-2.02, −1.58], *P* < .00001]. By further analysis of the common characteristics of the basic inclusion characteristics of the 2 studies we found that the duration of treatment in both studies was 4 weeks and that the medication used in the treatment group included *Bifidobacterium tetrasporium* tablets.

### 3.13. Anaplasma

**Figure 16.**
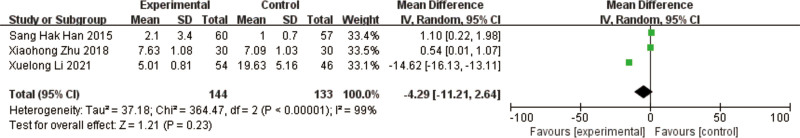
Forest plot for comparison of anaplasma between experimental group and control group. A total of 3 studies reported the mean change from baseline in anaplasma: the analysis showed that anaplasma levels were signifcantly reduced after probiotic intervention. The results were signifcantly different.

A total of 3 studies^[12,13,17]^ reported on anaplasma, with a total of 277 patients. The results of the heterogeneity test between the studies showed a large heterogeneity (*P* < .00001, *I²* = 99%), using a random effects model. The results of the meta-analysis showed a decrease in the abundance of *Methanobacterium* in the test group compared to the control group, with a nonsignificant difference in the results [MD = -4.29, 95% CI [-11.21, 2.64], *P* = .23] (Fig. 16).

The results of heterogeneity among studies were large, and to find out the reason of heterogeneity, by excluding one by one comparison, it was found that after excluding Xuelong Li et al,^[17]^ the results of study heterogeneity were low, I² = 12%, *P* = .29, and the results of meta-analysis showed that there was an increase in the experimental group of *B mimicus* compared to the control group, and the difference in results was significant [MD = 0.71, 95% CI [0.20, 1.21], *P* = .006].

### 3.14. TC

**Figure 17.**
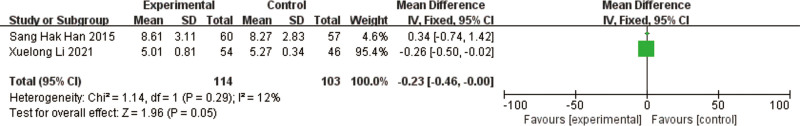
Forest plot of TC comparison between experimental group and control group. A total of 2 studies reported the mean change from baseline in TC: the analysis showed that TC levels were signifcantly reduced after probiotic intervention. The results were signifcantly different. TC = total cholesterol.

A total of 2 studies^[12,17]^ reported TC in a total of 154 patients. Meta-analysis showed that TC was reduced in the trial group compared to the treatment control group, with a significant difference in the results [MD = -0.23, 95% CI [-0.46, -0.00], *P* = .05] (Fig. 17).

### 3.15. Adverse effects

**Figure 18.**
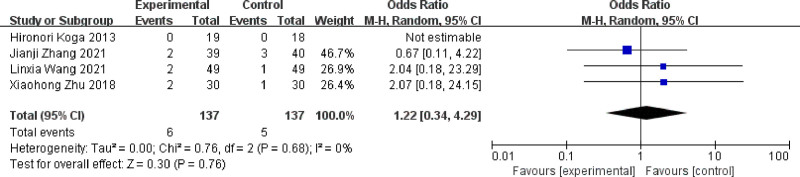
Forest plot for comparison of adverse reactions between experimental and control group. A total of 4 studies reported the adverse reactions: the analysis showed that adverse reactions were lightly lower in the posttreatment trial group compared to the posttreatment control group, with a nonsignificant difference in the results.

A total of 4 studies^[11,13,15,18]^ reported adverse effects, 274 patients in total. The results of the test for heterogeneity between studies showed a small heterogeneity (*P* = .68, I² = 0%), using a fixed-effects model. The results of the meta-analysis showed that the adverse reactions were slightly lower in the posttreatment trial group compared to the posttreatment control group, with a nonsignificant difference in the results [odds ratio = 1.22, 95% CI [0.34, 4.29], *P* = .76] (Fig. 18).

### 3.16. Course effect analysis

Because studies reported conducting different courses of treatment, we performed a subgroup analysis of ALT and AST reports from studies with different courses of treatment. A total of 3 studies,^[11,16,18]^ with a total of 212 patients, underwent a 4-week course of treatment and reported ALT. Meta-analysis showed that ALT was reduced in the trial group compared with the control group, with a significant difference [MD = -1.37, 95% CI [-2.50, -0.24, *P* = .02]. A total of 2 studies,^[13,17]^ with a total of 160 patients, underwent an 8-week course of treatment and reported ALT. Meta-analysis showed that ALT was reduced in the trial group compared to the control group, with a significant difference [MD = -1.12, 95% CI [-2.40, 0.15, *P* = .08]. A total of 3 studies,^[10,14,15]^ with a total of 244 patients, underwent a 12-week course of treatment and reported ALT. The results of the test for heterogeneity between studies showed a large heterogeneity (*P* < .00001, I² = 91%), using a random effects model. The results of the meta-analysis showed a reduction in ALT in the trial group compared to the control group, with a significant difference in the results [MD = -1.85, 95% CI [-2.90, -0.79, *P* = .0006].

The heterogeneous results between studies were large, and to identify the cause of the heterogeneity, an exclusion-by-exclusion comparison revealed that no studies with improved heterogeneous results were found in both the 4-week course and the 12-week course.

A total of 3 studies,^[11,16,18]^ with a total of 212 patients, underwent a 4-week course of treatment and reported AST. The results of the heterogeneity test between the studies showed a large heterogeneity (*P* < .00001, I² = 96%), using a random effects model. The results of the meta-analysis showed a reduction in ALT in the trial group compared to the control group, with a significant difference in the results [MD = -18.68, 95% CI [-32.92, -4.44, *P* < .00001]. A total of 1 study,^[17]^ with a total of 100 patients, performed 8 weeks of treatment and reported AST, showed a reduction in AST in the posttreatment trial group compared to the posttreatment control group, with a nonsignificant difference [MD = -14.10, 95% CI [-20.25, −7.95], *P* < .00001]. A total of 3 studies,^[10,14,15]^ with a total of 242 patients, underwent a 12-week course of treatment and reported AST. The results of the test for heterogeneity between studies showed a large heterogeneity (*P* = .04, I² = 69%), using a random effects model. Meta-analysis showed a reduction in AST in the treated trial group compared to the treated control group, with a significant difference in results [MD = -22.35, 95% CI [-26.40, −18.30], *P* < .00001].

The results of heterogeneity among studies were large, and to find out the reasons for the heterogeneity, by excluding one by one comparison, it was found that among the 4-week studies, 2 studies, Yuxia Zhou et al^[16]^ and Jianji Zhang et al,^[18]^ had low heterogeneity, I² = 0%, *P* = .45, and the results of meta-analysis showed that the test group had improved AST compared with the control group, and the difference in results was significant [MD = -26.21, 95% CI [-29.48, −22.94], *P* < .00001]. In the 12-week study, the heterogeneity was less after excluding Zhang Jianji et al^[15]^ (*P* < .00001, I² = 14%), and the results of the meta-analysis showed that the AST improved in the test group compared with the control group, with a significant difference in the results [MD = -20.64, 95% CI [-23.35, −17.92], *P* < .00001].

### 3.17. Publication bias

**Figure 19.**
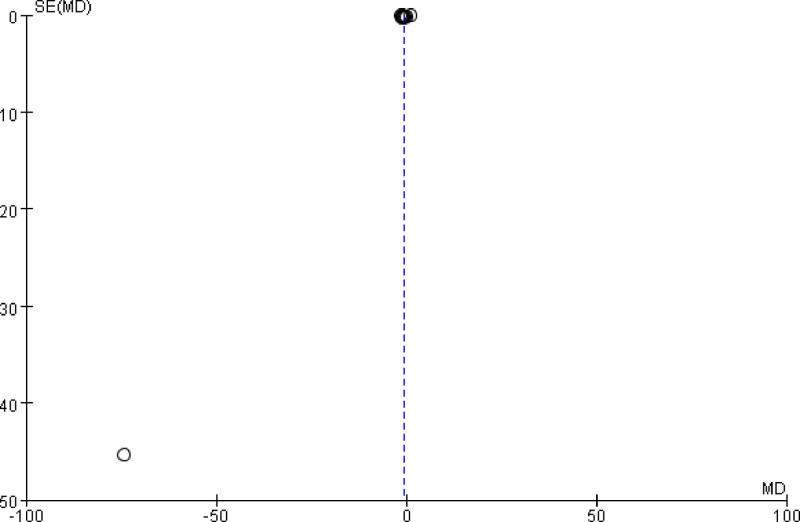
Funnel plot of *E coli* bias. The left and right scatter of *E coli* funnel plot results were basically symmetrical, with a low potential risk of publication bias.

Only one outcome indicator, *E coli*, was included in ≥ 10 papers in this study and the left and right scatter of *E coli* funnel plot results were basically symmetrical, with a low potential risk of publication bias. See Figure 2 for details Figure 19.

### 3.18. GRADE evidence grading

The GRADE evidence grading results showed that ALT, AST, GGT, TC, TBIL, h-CRP, TNF-α, IL-6, *E coli*, *Bifidobacterium*, *Lactobacillus*, *Enterococcus*, and adverse reactions were moderate quality evidence and IL-1β and *Bacteroidetes* were low quality evidence (Table 3).

**Table 3:** Probiotics and probiotics combined with other therapies for ALD outcome indicators of GRADE evidence score scale.

	Evaluation of the quality of evidentiary information							Sample size			
Closing indicators	Number of included studies	Type of research	Risk of bias	Inconsistency	Accuracy	Indirectness	Other	Test group	Control group	Quality	Importance
ALT	9^[10–19]^	RCT	Not serious	Serious*	Not serious	None	None	370	363	Medium	Key
AST	8^[10–12.14-18]^	RCT	Not serious	Serious*	Not serious	None	None	340	333	Medium	Key
GGT	9^[11–13,15–18]^	RCT	Not serious	Serious*	Not serious	None	None	340	340	Medium	Key
TC	2^[11.17]^	RCT	Not serious	Not serious	Serious†	None	None	78	76	Medium	Imp
TBIL	6^[10-12.14.17]^	RCT	Not serious	Serious*	Not serious	None	None	204	196	Medium	Imp
h-CRP	4^[11.12.15.17]^	RCT	Not serious	Not serious	Serious†	None	None	181	171	Medium	Imp
TNF-α	4^[12.15.17.19]^	RCT	Not serious	Serious*	Not serious	None	None	214	203	Medium	Imp
IL-1β	3^[12.14.17]^	RCT	Not serious	Serious*	Serious†	None	None	154	143	Low	Imp
IL-6	6^[11.12.14.17.18.19]^	RCT	Not serious	Serious*	Not serious	None	None	272	262	Medium	Imp
*E coli*	10^[10–19]^	RCT	Not serious	Serious*	Not serious	None	None	421	414	Medium	Imp
*Bifidobacteria*	^9[10.11.13–19]^	RCT	Not serious	Serious*	Not serious	None	None	361	357	Medium	Imp
*Lactobacillus*	8^[10.11.14.15.17–19]^	RCT	Not serious	Serious*	Not serious	None	None	269	273	Medium	Imp
*Enterococcus*	9^[10.12–19]^	RCT	Not serious	Serious*	Not serious	None	None	403	395	Medium	Imp
*Mycobacterium avium*	3^[12.13.17]^	RCT	Not serious	Serious*	Not serious	None	None	144	133	Low	Imp
Adverse reactions	4^[11.13.15.18.]^	RCT	Not serious	Not serious	Serious	None	None	137	137	Medium	Imp

ALD = alcoholic liver disease, ALT = alanine aminotransferase; AST = aspartate aminotransferase; GGT = γ-glutamyl transferase; h-CRP = high-sensitivity C-reactive protein; IL-1β = interleukin-1β; IL-6 = interleukin-6; Imp = important; RCT = randomized controlled trial; TBIL = total bilirubin; TC = total cholesterol; TNF-α = tumor necrosis factor-α.

* Large heterogeneity among groups.

† Small sample size.

## 4. Discussion

Currently, ALD is treated mainly by withdrawal therapy, and the only drugs recommended for the treatment of ALD according to EASL guidelines are glucocorticoids,^[20]^ but their treatment is affected by the duration of the disease as well as patient tolerance, and they cannot be used as long-term therapeutic drugs.^[21]^ Liver transplantation can only be used as a final remedy for the ineffectiveness of pharmacological treatment of ALD. Since the introduction of the gut–liver axis, more and more researchers have focused their attention on the intestinal microecology in the treatment of ALD.^[22]^ When intestinal microbes and their metabolites enter the liver through the portal vein, they activate IL-1β and its downstream pathways NOD-like Receptor Protein and caspase-1, increasing inflammatory factor production, enhancing hepatocyte sensitivity to death signals, and inducing steatosis.^[23]^ It has been found that pathogenic flora in the intestine of patients with ALD is significantly increased and metabolites of flora such as short-chain fatty acids are decreased.^[24,25]^ Therefore, the use of probiotics to regulate intestinal flora to improve ALD has become an important tool in the treatment of ALD. However, most of the current studies are based on animal experiments, and few clinical studies have been reported, and there is a wide variety of probiotics, and there is no uniform standard, so it is especially important to systematically evaluate the effectiveness and safety of probiotics or probiotic combination to improve ALD. Therefore, the use of probiotics to regulate intestinal flora to improve ALD has become an important tool in the treatment of ALD. However, most of the current studies are based on animal experiments, and few clinical studies have been reported, and there is a wide variety of probiotics, and there is no uniform standard, so it is especially important to systematically evaluate the effectiveness and safety of probiotics or probiotic combination to improve ALD.

In this study, a total of 10 studies on probiotics for the treatment of ALD were included after a comprehensive search, and ALT, AST, GGT, TC, TBIL, h-CRP, IL-6, IL-1β, TNF-α, *E coli*, *Bifidobacterium*, *Lactobacillus*, *Enterococcus*, and *Bacteroides* were used as efficacy indicators. The results of meta-analysis showed that probiotics or probiotic combination drugs were better than the conventional treatment, and adverse effects were used as safety indicators for systematic evaluation. The results of meta-analysis showed that probiotics or probiotic combination drugs were better than the conventional treatment group or placebo group in improving the outcome indicators such as ALT, AST, GGT, TC, TBIL, h-CRP, IL-6, IL-1β, TNF-α, *E coli*, *Bifidobacterium*, *Lactobacillus*, *Enterococcus*, *Synechococcus*, but the safety was not significantly different. It is suggested that the use of conventional therapy in combination with probiotic therapy may be considered by clinicians. However, the available clinical data are small and of average quality, and it is recommended that a large sample, high-quality randomized controlled trial of probiotic therapy in combination with ALD may be considered by clinicians. However, the available clinical data are small and of average quality, and it is recommended that a large sample, high-quality randomized controlled trial be conducted to provide scientific data support.

There are some limitations of this study: (1) a total of 10 papers were included in this study, which is a small sample size and has some influence on the reliability of the final results. (2) The overall quality of the included literature is average, one of the studies was not blinded, and all studies were explicitly assigned whether to hide, so there may be bias in the results. (3) There is a large clinical heterogeneity in the results because the probiotic species are not uniform, and some of the studies are probiotics combined with other treatments, and some of the control groups use placebo and some use pharmacological interventions. (4) Because the intestinal flora is affected by age, dietary conditions, geographical conditions, and the study results. The study results may have some bias.

In conclusion, probiotics or probiotics combined with drugs for ALD are superior to conventional treatment in improving liver. In conclusion, lipids, inflammation, and intestinal flora, and are expected to be a new effective treatment modality. However, limited by the number and quality of included studies, the conclusion still needs to be validated by more high-quality, multisample, and multicenter studies.

## Author contributions

**Data curation:** Xiangyu Zhou.

**Formal analysis:** Xiangyu Zhou.

**Funding acquisition:** Xiangyu Zhou.

**Project administration:** Xiangyu Zhou.

**Resources:** Xiangyu Zhou.

**Supervision:** Shuxi Zhou.
